# Who is Left Behind? Altruism of Giving, Happiness and Mental Health during the Covid-19 Period in the UK

**DOI:** 10.1007/s11482-020-09900-8

**Published:** 2020-12-17

**Authors:** Eleftherios Giovanis, Oznur Ozdamar

**Affiliations:** 1 Department of Economics, Policy and International Business (EPIB), Manchester Metropolitan University, Business School, All Saints, All Saints Campus, Manchester, M15 6BH UK; 2 Nazilli Faculty of Economics and Administrative Sciences, Department of Public Finance, Adnan Menderes University, Cumhuriyet, 09800 İsabeyli/Nazilli/Aydın, Turkey; 3 Faculty of Economics and Administrative Sciences, Department of Economics, Izmir University of Bakircay, Menemen, İzmir, Turkey

**Keywords:** Altruism, Covid-19, Difference-in-differences, Happiness, Money transfer, Subjective mental well-being

## Abstract

The UK government has decided to implement lockdown measures at the end of March 2020 as a response to the outbreak and spread of the Covid-19 pandemic. As a consequence, households have experienced job losses and a significant drop in their finances. During these unprecedented and difficult times, people provide financial assistance to those who are in need and have to cope with falls in their living standards. In this study we are interested to investigate the subjective well-being, which is expressed by mental health and components of general happiness, of the givers rather than of receivers. We apply a difference-in-differences framework to investigate the impact of altruism on the givers’ SWB in the UK. Altruism is denoted by transfers made to adult children, parents, siblings, and friends. Using the DiD estimator and the estimated coefficient of the household income we calculate the implicit willingness-to-pay (WTP) for altruism. We perform various regressions by gender and racial-ethnic background using data from the UK Household Longitudinal Study (UKHLS). The analysis shows that altruistic behaviours impact different domains of SWB between men and women, as well as, among people with different racial-ethnic background.

## Introduction

Altruism occurs in various ways, such as when we donate blood and to a charity or when we volunteer at a homeless shelter. There are several acts that happen all around us every day revealing people’s helping and altruistic behaviour. Some actions may represent a genuine altruism, while other behaviours are driven by self-concern. There are also moments when people do not help at all, seeming that they may not care for other people’s needs. Furthermore, reciprocal altruism (Trivers 1971) is very common, which is the belief that people support others, because they expect they will return their favour, should they need their support in the future. Hence, this may improve the chances of survival and reproductive success of givers, by supporting others, but also increases the chances of the receivers’ survival. According to Lishner and Stocks (2016), altruism is defined by two concepts; a motivation and a helping behaviour, suggesting that altruism can be described as a motive to enhance other people’s health and well-being. Klein and Dollenmayer (2014) define altruism as providing benefits to others at the givers’ risk of cost, and they argue that as long as there are costs, it is an act of altruism, including different motivations for kindness, such as happiness, compassion and pleasure.

The aim of this study is to explore whether the altruistic behaviours during the Covid-19 period had an impact on individuals’ SWB in the UK. While it is well documented that altruistic behaviours typically improve the recipient’s welfare and well-being at the cost of the giver’s energy and resource, little is known about the impact of altruistic acts on the subjective well-being (SWB) of the giver or performer (Harbaugh 1998; Post 2005; Dunn et al. 2008), especially during recession periods. People tend to provide financial and non-financial support to their family members and friends and this behaviour is important both from an economic and social perspective. Transfers are argued to be important in determining capital accumulation in transferring wealth to family members (Kotlikoff and Summers 1981; Cox and Raines 1985), and serving as a form of insurance against income shocks (Kotlikoff and Spivak 1981; Altonji et al. 1997). Furthermore, according to Bengtson and Roberts (1991) support to family and friends creates cohesion and solidarity among the social networks. The role of private transfers and the impact on family life has attracted the attention of researchers from various disciplines, including psychologists, economics and anthropologists. While their views differ depending on the discipline of the research, their common explanation given on the motivation of the private transfers fall within the two distinct categories we mentioned earlier: altruism and self-interest (Trivers 1971; Berkowitz 1972; Becker 1976; Batson 1991; Cox and Rank 1992; Khalil 2004).

Furthermore, even though there are studies exploring the impact of altruistic acts on mental health and happiness, to the best of our knowledge there is no study exploring the impact of altruism and evaluating its value in various components of SWB and happiness, such as confidence, capability of making decisions, whether the respondent feels constantly under strain or plays a significant role in the family and the society. Using the Covid-19 lockdown period as an exogenous shock we aim to investigate how the altruistic behaviour- expressed by transfers made to different members of the family and friends- varies by the recipient, as well as, by the giver’s gender and racial background. We apply a standard difference-in-differences (DiD) framework, where the treated group includes the givers and the control group includes the non-givers. Following the discussion so far, we perform regressions by gender and racial-ethnic background. The empirical analysis relies on data from the UK Household Longitudinal Study (UKHLS) during the period 2015 and the April of 2020. Next, we use a similar technique to the well-known Life Satisfaction Approach (LSA) to calculate the marginal willingness to pay (WTP), which shows how much individuals behave altruistically to improve their SWB (see Frey et al. 2010 for an example of the LSA). We find a large heterogeneity across gender and racial-ethnic groups not only in the amount of the marginal willingness to pay, but also in the impact of altruism and transfers made in different domains of the SWB.

## Literature Review

One of the earliest studies about altruism and volunteering, by Hunter and Linn (1980–1981), compared the retirees older than 65 years who volunteered with those who did not, while both groups were sharing the same demographic and other background characteristics. The authors found that the former group reported significantly higher levels of life satisfaction and presented fewer symptoms of anxiety and depression. In another study, families deciding to donate their organs experienced psychological benefits (Batten and Prottas 1987). More recent studies also confirm the association between altruistic activities, mental health and SWB (Krueger et al. 2001; Liang et al. 2001; Dulin and Hill 2003; Musick and Wilson 2003; Morrow-Howell et al. 2003).

Studies suggest that humans have not altruistic desires (Cialdini et al. 1997), they are naturally egoistic and thus, genuine altruism is impossible, other studies found that people exhibit altruistic behaviour (Batson 1991; Stich et al. 2010), while recent research indicates that altruistic behaviours are reflexive, intuitive and automatic (Zaki and Mitchell 2013). Researchers looked at the reasons behind the helping actions and altruistic behaviours and used various theories trying to understand and explain the motive for such behaviour, but there is no definitive answer yet (Batson et al. 2003). There is a long and well-documented literature developing various theories trying to explain the altruistic behaviours. One popular theoretical model is the arousal-reduction model, and according to that, people may experience a state of arousal when they observe an emergency. As this state of arousal, which can be expressed by anxiety and distress, increases, it becomes more unpleasant. Hence, to reduce the arousal, people are responding to this emergency (Piliavin et al. 1982). Another model is the negative-state relief explanation, which states that people witnessing another person being in distress, feel empathy and try to help that person that also allows to avoid the experience of negative emotions, such as shame and guilt (Cialdini et al. 1987). The Social Learning Theory (SLT) developed by Bandura (1977), states that people become socialized and can learn to associate rewards and punishment with helping behaviour. According to Bandura, people are constantly processing information and learn to consider the consequences of their actions.

Cox and Stark (1994) have also explored the hypothesis of “demonstration effect” as one of the main reasons for financial transfers between parents and children, arguing that other theories of intergenerational transfers may not always explain the motives of such transfers. In particular, if motives, such as the exchange or rewarding have only a mild effect on the children’s behaviour, the authors introduce the idea of “preference shaping”, arguing that parents behave in such a way and are involved in transfers to reinforce and secure support from their offspring generations. Nevertheless, our study differs, as it explores the relationship between transfers and the giver’s SWB, rather than of the recipient. Various studies have explored the impact of altruism on the mental health and well-being of providers. However, we exploit the Covid-19 pandemic to investigate how people behave in such periods of shocks and how the transfers influence the well-being by gender and ethnic background. Furthermore, our results show whether and how givers support multiple family members and friends (Dulin and Hill 2003; Schwartz et al. 2003; Peterson 2006).

## Data and Methods

### Data

For the empirical specification we derive the data from the UK Household Longitudinal Study (UKHLS), which is a nationally representative survey of approximately 40,000 households started in 2009 and is administered by the Institute for Social and Economic Research (ISER) at the University of Essex. There are currently 9 waves and for the pre-Covid 19 lockdown period we use the waves 7–9 covering the period 2015–2019. For the Covid-19 period we use a special instance of the UKHLS survey conducted in April 2020, which is designed to explore the effects of the Covid-19 pandemic crisis. The survey provides a rich information about the financial and employment situation, housing caring responsibilities, transfers made to family, relatives and friends, and mental well-being measures.

Our estimates remain robust if we consider a shorter pre-Covid period, such as the wave 8 and 9. Nevertheless, we prefer to obtain also wave 7 to test for the parallel trend assumption and the DiD validity. We explore various subjective well-being (SWB) measures. The first is the 12 item General Health Questionnaire caseness score (GHQ-12), which is a well-documented and prominent measure in academic research. GHQ-12 is a multidimensional scale that assesses several distinct aspects of mental distress and it is significantly correlated with measures of depression, happiness and self-esteem (Tait et al. 2003; Del-Pilar Sánchez-López and Dresch 2008; Romppel et al. 2013) The GHQ-12 takes values between 0, implying an excellent psychological well-being, to 12 that indicates very poor well-being.

The second measure is the general happiness and its various components. In particular, the first set of components explored are: the overall happiness; concentration; playing a useful role; capable of making decisions; enjoy day-to-day activities and ability to face problems. Possible answers are four and more specifically are: “More so than usual”, “About the same as usual”, “Less so than usual” and “Much less than usual”. The second set of the remained components answer as: “Not at all”, “No more than usual”, “Rather more than usual” and “Much more than usual” and these include: constantly under strain; depressed; problem overcoming difficulties; losing confidence; believe that the respondent is worthless and loss of sleep. In all cases, the components are measured on a Likert scale from 1 to 4, based on the answers we mentioned above, with higher values associated with lower levels of well-being.

### Empirical Specification

The aim of this study is to investigate the impact of transfers made to different persons, family members and friends, due to Covid-19 and the lockdown measures, on the givers’ mental health measured by the GHQ and the components of happiness. We propose the following difference-in-differences (DiD) strategy:

1 $$ {SWB}_{i,r,t}={\beta}_0+{\beta}_1{TM}_{i,r,t}+{\beta}_2 covid{19}_{i,r,t}+{\beta}_3\left({TM}_{i,r,t}\cdotp covid{19}_{i,r,t}\right)+{\beta}_4\log \left({y}_{i,r,t}\right)++{\beta}^{\prime }{\boldsymbol{X}}_{i,r,t}+{\theta}_t+{l}_r+{u}_{i,r,t} $$

Where *SWB* denotes the subjective well-being for individual *i* in region *r*, and at time-wave *t*. Variable *TM* denotes the transfers made from the givers to four main groups: adult children; parents and grandparents; siblings and friends. *Log(y)* denotes the logarithm of the monthly household income expressed in prices of 2019 and the average value is around £4155. Set *l*_*r*_ indicates the area-government region fixed effects, time dummies expressed by set *θ*_*t*_. Parameter *β*_*3*_ is the DiD estimator that identifies the effect on the outcome variables of the transfers made compared to those who did not made any transfer. Based on the data availability the control variables in vector **X** include gender, age, employment status, whether there are children in the household, and dummies for the month and the year of the interview.

We estimate model (1) using the ordinary least squares (OLS), accounting for the weight of the survey design in order to avoid biased statistical inference and sample attrition (Chen et al. 2015). Furthermore, we cluster the standard errors at the individual level. We limit the sample only to those that have non-missing values and the analytical sample is 2568 individuals. In particular, we follow the same 2568 individuals across the 4 waves which results to 10,272 observations (2568 × 4). Therefore, we prefer to have a balanced panel data, where we include in our empirical analysis only those who are observed and followed in all 4 waves of the survey. In other words, we limit the analysis to those who replied in the question on whether they have made the transfers we explore during the pandemic period (treated group) and those who have not (control group).

Next, we will estimate the marginal *WTP* of well-being, and this will reveal how much money should be allocated to compensate people for experiencing higher levels of SWB due to altruism acts during the Covid-19 lockdown period. Using the LSA this can be found as:

2 $$ WTPA= dy/ dx= dlog(y)/ dDiD=\left(\partial SWB/\partial DiD\right)/\Big(\partial SWB/\left(\partial\ \log (y)\right) $$

Where *WTPA* denotes the marginal willingness to pay of altruism that improves the SWB and is invariant to any monotonic transformation of function (1), as no cardinal utility function is required (Frey et al. 2010). The *dy* and *dx* are the first derivatives of the theoretical function. In particular, it will be the first derivative of the SWB function with respect to the DiD estimator, which is the coefficient *β*_*3*_ in Eq. (1), over the first derivative of the SWB function with respect to the logarithm of the monthly household income, represented by the coefficient *β*_*4*_. While we use the LSA, we do not further discuss this approach, since it has been extensively used in the literature. More details about its advantages and limitations compared to the hedonic price analysis and the contingent evaluation techniques can be found in earlier studies (e.g. Frey et al. 2010; Levinson 2012; Giovanis 2019). Moreover, we have estimated the regressions using the ordered Logit model and the Fixed Effects-OLS models, and the marginal effects and *WTP* were found to be very close to those derived by the OLS, which is also supported by earlier studies (e.g. Ferrer-i-Carbonell and Frijters 2004).

We will test for the parallel trend hypothesis estimating a DID model using lags of the treated group as (see Angrist and Pischke 2008 for more details):

3 $$ {SWB}_{i,r,t}={\sum}_{j=q}^n{\beta}_j{DiD}_{i.r.t-j}+{\beta}_1\log (y)+{\beta}^{\prime }{\boldsymbol{X}}_{i,r,t}+{\theta}_t+{l}_r+{u}_{i,r,t} $$

Where *DiD*_*i,r,t*_ is the *DiD* estimator showing whether the treatment-Covid-19 lockdown is switched on in year *t*, and the lags of the treatment are expressed respectively by *q* for *n* = 1,…,4 corresponding to years 2016–2019. We will perform a joint hypothesis testing for the DiD lagged coefficient, and the null hypothesis implies that the parallel trend assumption holds. Regression (3) could have included also leads, but since we have only one post-shock period we cannot implement a test including both leads and lags. Furthermore, we do not include the DiD lagged value in year 2015, as this is dropped due to multicollinearity.

## Results

### Estimates by Total Sample

In Table 1 we report the estimates of the DiD design using the full sample. While in the previous section we have mentioned that we will use all the SWB measures, we report only those where we have found a significant DiD coefficient. Hence, in Table 1 and regarding the transfers made to adult children, we find the parameter *β*_*3*_ is significant in the *GHQ-12 Caseness*; *playing a useful role*; *losing confidence*; *believe worthless* and *happiness* regressions. The *WTPA* is around £160–170 in the *GHQ-12* and *believe worthless* regressions, and it reaches the £350 and £420 respectively in the *losing confidence* and *happiness* regressions and £730 in the regression of *playing a useful role.* Thus, the DiD estimates show us how much individual’s well-being is improved by altruistic behaviours, expressed by transfers made, during the Covid-19 lockdown period.

**Table 1:** DiD estimates for transfers made-total sample

Treated (Transfers Made to Adult Children)		Treated (Transfers Made to Parents-Grandparents)	
GHQ-12 Caseness		Playing a Useful Role	
Transfer Made* Covid-19 Period	−0.1408* (0.0795)	Transfer Made* Covid-19 Period	−0.0670** (0.0332)
Logarithm of Monthly Household Income	−0.4540*** (0.0725)	Logarithm of Monthly Household Income	−0.0669*** (0.0137)
MWTP Altruism	£160	MWTP Altruism	£480
No. Observations	10,272	No. Observations	10,272
R-Square	0.0750	R-Square	0.0285
Pre-treatment F-Statistic Test	2.564 [0.2774]	Pre-treatment F-Statistic Test	2.317 [0.3139]
Playing a Useful Role		Capable of Making Decisions	
Transfer Made* Covid-19 Period	−0.0995** (0.0469)	Transfer Made* Covid-19 Period	−0.0762** (0.0384)
Logarithm of Monthly Household Income	−0.0670*** (0.0136)	Logarithm of Monthly Household Income	−0.0425*** (0.0097)
MWTP Altruism	£730	MWTP Altruism	£750
No. Observations	10,272	No. Observations	10,272
R-Square	0.0291	R-Square	0.0188
Pre-treatment F-Statistic Test	0.0777 [0.9619]	Pre-treatment F-Statistic Test	2.911 [0.2333]
Losing Confidence		Enjoy day-to-day Activities	
Transfer Made* Covid-19 Period	−0.0784** (0.0383)	Transfer Made* Covid-19 Period	−0.0757* (0.0419)
Logarithm of Monthly Household Income	−0.1126*** (0.0176)	Logarithm of Monthly Household Income	−0.0484*** (0.0134)
MWTP Altruism	£350	MWTP Altruism	£680
No. Observations	10,272	No. Observations	10,272
R-Square	0.0718	R-Square	0.0613
Pre-treatment F-Statistic Test	2.572 [0.2764]	Pre-treatment F-Statistic Test	0.6596 [0.7591]
Believe worthless		Depression	
Transfer Made* Covid-19 Period	−0.0416** (0.0202)	Transfer Made* Covid-19 Period	−0.0941* (0.0547)
Logarithm of Monthly Household Income	−0.1224*** (0.0161)	Logarithm of Monthly Household Income	−0.1088*** (0.0186)
MWTP Altruism	£170	MWTP Altruism	£440
No. Observations	10,272	No. Observations	10,272
R-Square	0.0681	R-Square	0.0603
Pre-treatment F-Statistic Test	0.1321 [0.9461]	Pre-treatment F-Statistic Test	0.1615 [0.9224]
Happiness			
Transfer Made* Covid-19 Period	−0.0371** (0.0188)		
Logarithm of Monthly Household Income	−0.0432*** (0.0128)		
MWTP Altruism	£420		
No. Observations	10,272		
R-Square	0.0213		
Pre-treatment F-Statistic Test	0.2354 [0.8891]		
Treated (Transfers Made to Siblings)		Treated (Transfers Made to Friends)	
GHQ-12 Caseness		GHQ-12 Caseness	
Transfer Made* Covid-19 Period	−0.3206** (0.1505)	Transfer Made* Covid-19 Period	−0.0595* (0.0311)
Logarithm of Monthly Household Income	−0.4498*** (0.0721)	Logarithm of Monthly Household Income	−0.4489*** (0.0718)
MWTP Altruism	£360	MWTP Altruism	£80
No. Observations	10,272	No. Observations	10,272
R-Square	0.0761	R-Square	0.0762
Pre-treatment F-Statistic Test	1.628 [0.4431]	Pre-treatment F-Statistic Test	1.302 [0.5115]
Playing a Useful Role		Ability to Face Problems	
Transfer Made* Covid-19 Period	−0.1008* (0.0525)	Transfer Made* Covid-19 Period	−0.0743** (0.0362)
Logarithm of Monthly Household Income	−0.0480*** (0.0171)	Logarithm of Monthly Household Income	−0.0320*** (0.0099)
MWTP Altruism	£980	MWTP Altruism	£1150
No. Observations	10,272	No. Observations	10,272
R-Square	0.0688	R-Square	0.0208
Pre-treatment F-Statistic Test	1.561 [0.4582]	Pre-treatment F-Statistic Test	1.129 [0.5686]
Depression		Believe worthless	
Transfer Made* Covid-19 Period	−0.1620** (0.0804)	Transfer Made* Covid-19 Period	−0.0671** (0.0328)
Logarithm of Monthly Household Income	−0.1109*** (0.0185)	Logarithm of Monthly Household Income	−0.1218*** (0.0160)
MWTP Altruism	£720	MWTP Altruism	£280
No. Observations	10,272	No. Observations	10,272
R-Square	0.0599	R-Square	0.0680
Pre-treatment F-Statistic Test	0.5284 [0.7678]	Pre-treatment F-Statistic Test	1.273 [0.5292]
Believe worthless		Happiness	
Transfer Made* Covid-19 Period	−0.1718*** (0.0640)	Transfer Made* Covid-19 Period	−0.0351** (0.0166)
Logarithm of Monthly Household Income	−0.1215*** (0.0152)	Logarithm of Monthly Household Income	−0.0421*** (0.0128)
MWTP Altruism	£700	MWTP Altruism	£420
No. Observations	10,272	No. Observations	10,272
R-Square	0.0698	R-Square	0.0217
Pre-treatment F-Statistic Test	0.0567 [0.9720]	Pre-treatment F-Statistic Test	0.1339 [0.9353]
Happiness			
Transfer Made* Covid-19 Period	−0.0428** (0.0211)		
Logarithm of Monthly Household Income	−0.0422*** (0.0126)		
MWTP Altruism	£510		
No. Observations	10,272		
R-Square	0.0223		
Pre-treatment F-Statistic Test	1.4322 [0.4982]		

Standard errors in the brackets and clustered at the individual level. P-values within the square brackets. ***, ** and * indicate significance at 1%, 5% and 10% level. Regressions are weighted by the sampling survey weight

The low *WTPA* values is due to the high coefficient of the household income, which seems to contribute significantly higher to the mental health, compared to the happiness components. Similarly, *playing a useful role* and *depression* are significant parts of the altruism when the transfers are made to parents and grandparents, with the most important component being *capable of making decisions*. Next, we report the estimates for the transfers made to siblings and those made to friends. We found similar SWB measures to be important in the individuals’ altruism, with the components of *playing a useful role*, *believe worthless* and *depression* being the most important regarding the transfers made to siblings, while the *ability to face problems* followed by *happiness* are found to be the altruistic behaviours contributing mostly in the SWB based on the values of the *WTPA*.

Overall, while we find some common altruistic behaviours in the four sets of transfers we explore, there are differences in terms of the *WTPA* and some components found to be significant in a set of transfers made and insignificant in some other sets. In particular, the component of “playing a useful role” and the *WTPA* value is found to be higher in the transfers made to adult children and siblings, while “capable of making decisions” and “ability to face problems” are found to be the most important respectively in the transfers made to parents-grandparents and friends.

**Fig. 1:**
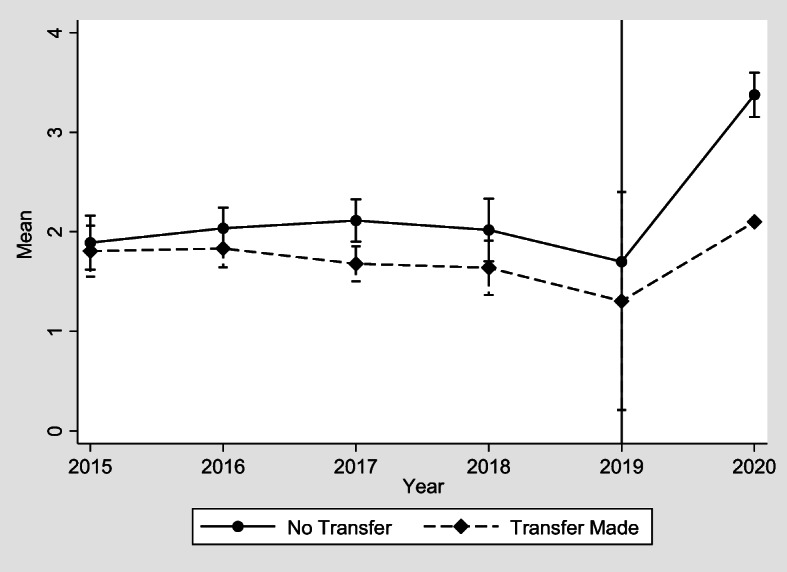
Transfers made to adult children and mental health GHQ-12

**Fig. 2:**
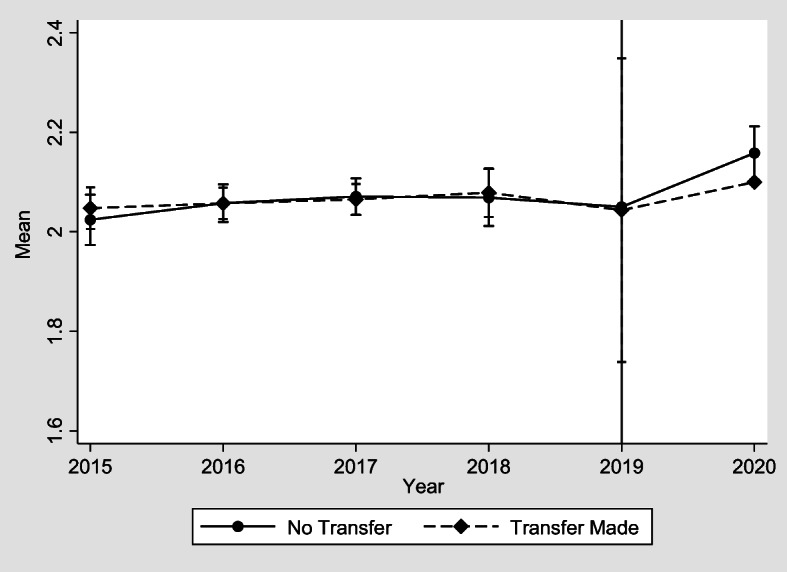
Transfers made to adult children and playing a useful role

**Fig. 3:**
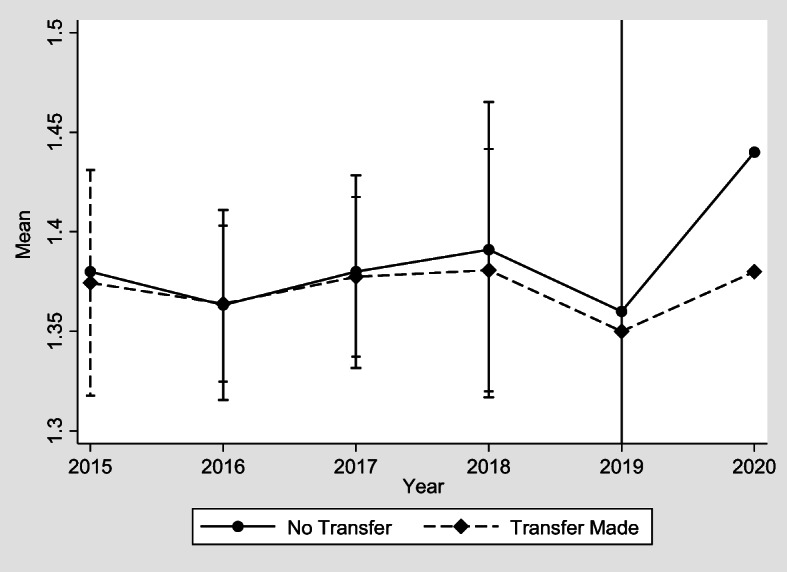
Transfers made to adult children and believe worthless

**Fig. 4:**
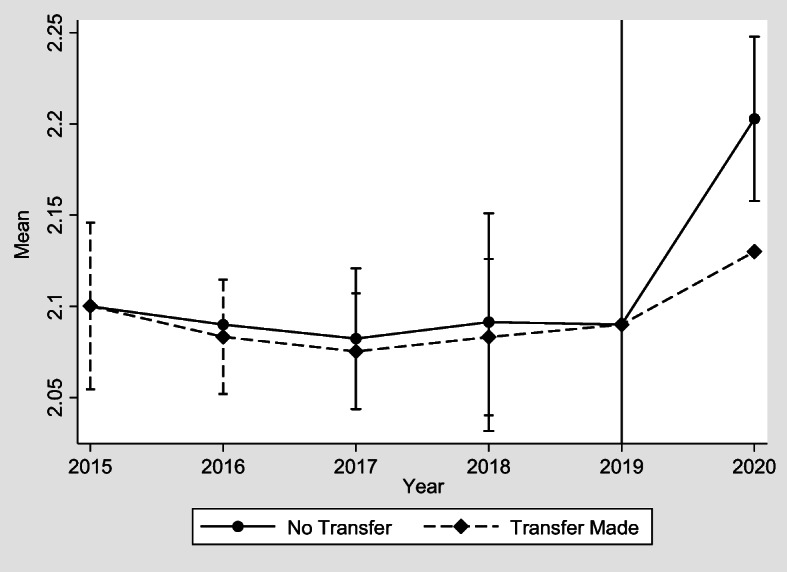
Transfers made to adult children and happiness

In Figs. 1, 2, 3 and 4 we illustrate the average values of four SWB measures used in Table 1 and for transfers made to adult children. While we do not present the graphs for the rest of the transfers made, we should note that we derive the same concluding remarks. In particular, we see that the parallel trend assumption appears to hold before he Covid-19 lockdown period, while a jump upwards is observed for both groups- those who made the transfers and those who did not- during the Covid-19 period, indicating that lockdown has affected negatively the SWB of both treated and control subjects. Nevertheless, we see a higher jump upwards for those who have not made the transfers, as it has been also shown in the results of Table 1. Furthermore, according to the pre-treatment *F-statistic* tests and the *p-values* we accept the null hypothesis, implying that the parallel trend assumption holds in all cases. In this case we test the joint significance of the DID estimated coefficients of regression (3) with 1–4 lags, corresponding to the years 2016–2019 in Figs. 1, 2, 3 and 4.

### Estimates by Gender

To offer more insights, in Table 2 we report the estimates by gender. Regarding the transfers made to adult children we see a large heterogeneity, as for males these transfers affect the mental health measured by the GHQ-12, losing confidence and whether they believe are less worthless, while for females, transfers affect their happiness, whether they play a useful role and enjoy daily activities. Regarding the transfers made to parents and grandparents, females report a reduction in their depression and improvement of joy in their daily activities, while males see an improvement in their capability of making decisions and playing a useful role.

**Table 2:** DiD estimates for transfers made by gender

Treated (Transfers Made to Adult Children)-MALE		Treated (Transfers Made to Adult Children)-FEMALE	
GHQ-12 Caseness		Playing a Useful Role	
Transfer Made* Covid-19 Period	−0.3721** (0.1824)	Transfer Made* Covid-19 Period	−0.1765*** (0.0645)
Logarithm of Monthly Household Income	−0.4134*** (0.1215)	Logarithm of Monthly Household Income	−0.0540*** (0.0178)
MWTP Altruism	£400	MWTP Altruism	£1200
No. Observations	4288	No. Observations	5984
R-Square	−3.4106	R-Square	0.0472
Pre-treatment F-Statistic Test	2.8822 [0.2367]	Pre-treatment F-Statistic Test	0.0804 [0.9606]
Losing Confidence		Enjoy day-to-day Activities	
Transfer Made* Covid-19 Period	−0.1346** (0.0679)	Transfer Made* Covid-19 Period	−0.1153** (0.0474)
Logarithm of Monthly Household Income	−0.1029*** (0.0281)	Logarithm of Monthly Household Income	−0.0372** (0.0167)
MWTP Altruism	£660	MWTP Altruism	£1350
No. Observations	4288	No. Observations	5984
R-Square	0.0694	R-Square	0.0727
Pre-treatment F-Statistic Test	0.1087 [0.9471]	Pre-treatment F-Statistic Test	0.8268 [0.6614]
Believe worthless		Happiness	
Transfer Made* Covid-19 Period	−0.0350* (0.0185)	Transfer Made* Covid-19 Period	−0.0482** (0.0223)
Logarithm of Monthly Household Income	−0.1098*** (0.0259)	Logarithm of Monthly Household Income	−0.0412*** (0.0122)
MWTP Altruism	£160	MWTP Altruism	£600
No. Observations	4288	No. Observations	5984
R-Square	0.0747	R-Square	0.0309
Pre-treatment F-Statistic Test	0.2637 [0.8765]	Pre-treatment F-Statistic Test	0.3054 [0.8584]
Treated (Transfers Made to Parents-Grandparents)-MALE		Treated (Transfers Made to Parents-Grandparents)-FEMALE	
Playing a Useful Role		Enjoy day-to-day Activities	
Transfer Made* Covid-19 Period	−0.1092** (0.0454)	Transfer Made* Covid-19 Period	−0.0916** (0.0451)
Logarithm of Monthly Household Income	−0.0725*** (0.0209)	Logarithm of Monthly Household Income	−0.0371** (0.0167)
MWTP Altruism	£760	MWTP Altruism	£1100
No. Observations	4288	No. Observations	5984
R-Square	0.0334	R-Square	0.0696
Pre-treatment F-Statistic Test	2.219 [0.3297]	Pre-treatment F-Statistic Test	0.0179 [0.9911]
Capable of Making Decisions		Depression	
Transfer Made* Covid-19 Period	−0.0571** (0.0284)	Transfer Made* Covid-19 Period	−0.1174** (0.0566)
Logarithm of Monthly Household Income	−0.0301** (0.0149)	Logarithm of Monthly Household Income	−0.1027*** (0.0232)
MWTP Altruism	£960	MWTP Altruism	£650
No. Observations	4288	No. Observations	5984
R-Square	0.0334	R-Square	0.0627
Pre-treatment F-Statistic Test	0.9377 [0.6257]	Pre-treatment F-Statistic Test	0.4281 [0.8074]
Treated (Transfers Made to Siblings) -MALE		Treated (Transfers Made to Siblings) –MALE	
GHQ-12 Caseness		Loss of Sleep	
Transfer Made* Covid-19 Period	−0.9481** (0.4511)	Transfer Made* Covid-19 Period	−0.2539** (0.1131)
Logarithm of Monthly Household Income	−0.4045*** (0.1219)	Logarithm of Monthly Household Income	−0.0693** (0.0299)
MWTP Altruism	£1180	MWTP Altruism	£1850
No. Observations	4288	No. Observations	4288
R-Square	0.0689	R-Square	0.0450
Pre-treatment F-Statistic Test	0.5164 [0.7724]	Pre-treatment F-Statistic Test	0.9783 [0.6131]
Concentration		Playing a Useful Role	
Transfer Made* Covid-19 Period	−0.2001** (0.0904)	Transfer Made* Covid-19 Period	−0.3034*** (0.1081)
Logarithm of Monthly Household Income	−0.0390** (0.0181)	Logarithm of Monthly Household Income	−0.0754*** (0.0208)
MWTP Altruism	£2600	MWTP Altruism	£2030
No. Observations	4288	No. Observations	4288
R-Square	0.0278	R-Square	0.0347
Pre-treatment F-Statistic Test	0.0567 [0.9720]	Pre-treatment F-Statistic Test	0.8203 [0.6635]
Depression			
Transfer Made* Covid-19 Period	−0.3532*** (0.1172)		
Logarithm of Monthly Household Income	−0.1111*** (0.0306)		
MWTP Altruism	£1600		
No. Observations	4288		
R-Square	0.0648		
Pre-treatment F-Statistic Test	3.515 [0.1725]		
Treated (Transfers Made to Siblings) -FEMALE			
Enjoy day-to-day Activities		Believe worthless	
Transfer Made* Covid-19 Period	−0.1423** (0.0668)	Transfer Made* Covid-19 Period	−0.1211** (0.0561)
Logarithm of Monthly Household Income	−0.1198*** (0.0201)	Logarithm of Monthly Household Income	−0.0325*** (0.0097)
MWTP Altruism	£590	MWTP Altruism	£1500
No. Observations	5984	No. Observations	5984
R-Square	0.0776	R-Square	0.0339
Pre-treatment F-Statistic Test	1.151 [0.5626]	Pre-treatment F-Statistic Test	0.7286 [0.6947]
Treated (Transfers Made to Friends)-MALE		Treated (Transfers Made to Friends)-FEMALE	
GHQ-12 Caseness		Playing a Useful Role	
Transfer Made* Covid-19 Period	−0.4150* (0.2296)	Transfer Made* Covid-19 Period	−0.1444** (0.0661)
Logarithm of Monthly Household Income	−0.3931*** (0.1205)	Logarithm of Monthly Household Income	−0.0552*** (0.0177)
MWTP Altruism	£540	MWTP Altruism	£1300
No. Observations	4288	No. Observations	5984
R-Square	0.0673	R-Square	0.0447
Pre-treatment F-Statistic Test	0.6012 [0.7404]	Pre-treatment F-Statistic Test	0.4135 [0.8132]
Playing a Useful Role		Believe Worthless	
Transfer Made* Covid-19 Period	−0.2398** (0.1149)	Transfer Made* Covid-19 Period	−0.0578** (0.0273)
Logarithm of Monthly Household Income	−0.0741*** (0.0207)	Logarithm of Monthly Household Income	−0.1206*** (0.0202)
MWTP Altruism	£1640	MWTP Altruism	£240
No. Observations	4288	No. Observations	5984
R-Square	0.0335	R-Square	0.0794
Pre-treatment F-Statistic Test	3.1251 [0.2096]	Pre-treatment F-Statistic Test	2.3541 [0.3082]
Believe worthless		Happiness	
Transfer Made* Covid-19 Period	−0.0823** (0.0407)	Transfer Made* Covid-19 Period	−0.0668** (0.0668)
Logarithm of Monthly Household Income	−0.1050*** (0.0254)	Logarithm of Monthly Household Income	−0.0375** (0.0165)
MWTP Altruism	£400	MWTP Altruism	£880
No. Observations	4288	No. Observations	5984
R-Square	0.0760	R-Square	0.0331
Pre-treatment F-Statistic Test	3.2161 [0.2003]	Pre-treatment F-Statistic Test	0.1251 [0.9394]

Standard errors in the brackets and clustered at the individual level. P-values within the square brackets. ***, ** and * indicate significance at 1%, 5% and 10% level. Regressions are weighted by the sampling survey weight

Numerous studies found that women give on average more than men in Dictator Game (DG) experiments (Eckel and Grossman 1998; Dickinson and Tiefenthaler 2002; Andreoni and Vesterlund 2001; Dufwenberg and Muren 2006; Houser and Schunk 2009; Dreber et al. 2013, 2014; Capraro et al. 2014; Capraro and Marcelletti 2014; Rand et al. 2016). However, there are critical exceptions, as Engel (2011) using a meta-analysis of 616 DG experiments found that women are only marginally significantly more altruistic than men.

Our results confirm the findings by Carpenter et al. (2008) and Cappelen et al. (2015) using the Dictator Game (DG), which have compared student samples to random samples and found gender differences in the student samples, but not in the random samples. This had led them to the conclusion that gender differences in DG altruism, if existing, may be domain specific, as we found that altruism affects different components of SWB. Hence, comparing the *WTPA* values we find differences across gender, as women are more likely to pay significantly higher for transfers made to adult children at £1350, which is the maximum *WTPA* value that refers to joy with daily activities, compared to the £660 for men willing to pay to improve their confidence. Furthermore, we find differences in the altruistic behaviour across gender and the SWB domains (Carpenter et al. 2008; and Cappelen et al. 2015). More specifically, men evaluate more the capability of making decisions and preservation of confidence, showing that men care more about their capability and control, while women evaluate more their depression, happiness and joy with day-to-day activities.

While Andreoni and Vesterlund (2001) try to investigate the role of price changes on altruistic behaviour, we aim to explore the gender differences across various domains of SWB. Regarding transfers to parents and grandparents the results also reveal differences in the altruistic behaviours between males and females, as the former group prefers to improve its role and the capability of making decisions, while altruism affects the joy with daily activities and depression of women, supporting the previous findings, that altruistic behaviours for women are related to happiness and warmth that helps with social bonding and is more consistent with the traditional role as primary caregiver (Plant et al. 2000). On the other hand, men display more contempt and pride, which is consistent with a provider and protector role (Plant et al. 2000), and is expressed from the capability of making decisions and feeling worthless.

Next we report the estimates for the transfers made to siblings and friends. Regarding siblings, we derive different conclusions compared to the previous findings. In particular, altruism affects males in a large number of SWB domains, including *loss of sleep*, *concentration*, *GHQ-12*, *depression* and *playing a useful role*. On the other hand, joy with daily activities and *feeling worthless* are the most important SWB domains for women. Furthermore, the *WTPA* in this case is higher in the male group with a maximum value at £2600 to improve the concentration, while for women the *WTPA* reaches the £1500 to reduce the feeling of being worthless during the Covid-19 lockdown period. Therefore, based on the *WTPA* values, women are more willing to pay and to offer support to adult children, parents and grandparents, while men are willing to support more their siblings. Finally, we present the estimates for the transfers made to friends, and we find similar impact of the altruistic acts in both sexes, as mental health, happiness and feeling worthless are common for both men and women.

There is a large literature in psychology trying to explain the gender differences in altruistic behaviour. For instance, according to Derntl et al. (2010) women appear to use more the emotional brain areas, while men use more reflective brain areas. In another study by Heintz et al. (2019), using a sample of more than one million women, it is found that women tend to express higher levels of benevolence-related character strengths, such as love, gratitude and kindness, which is revealed by the significant impact of altruism on happiness, depression and joy with daily activities in our analysis.

### Estimates by Racial and Ethnic Background

In Table 3 we report the estimates across the individuals’ racial and ethnic background. We should notice that the estimates for the white British are very close with those found in Table 1, since this group consists almost of the 75% of the sample. Overall, according to the *WTPA* values, we find that individuals value more the transfers made to friends and siblings. Furthermore, the altruistic behaviour by the recipient type, as the *GHQ-12*, *happiness*, *depression*, *losing confidence* and *feeling worthless* are mainly influenced by the transfers made to adult children and parents-grandparents, while the *ability to face problems* and *overcome difficulties* are also impacted from transfers made to siblings and friends. The findings for the non-UK born whites and mixed whites (e.g. white and black or white and Asian) are very similar regarding the domains of SWB influenced by the transfers made to various groups of recipients, except for transfers made to adult children, which are not reported in Table 3, since the DiD estimators were found insignificant. On the other hand, based on the *WTPA*, other whites and mixed whites value more the SWB derived from transfers made to parents-grandparents and siblings than friends as we found for the white British.

**Table 3:** DiD estimates for transfers made by race and ethnicity

Treated (Transfers Made to Adult Children)-White British		Treated (Transfers Made to Parents-Grandparents)- White British	
GHQ-12 Caseness		Capable of Making Decisions	
Transfer Made* Covid-19 Period	−0.2310** (0.1143)	Transfer Made* Covid-19 Period	−0.0720* (0.0359)
Logarithm of Monthly Household Income	−0.4140*** (0.0764)	Logarithm of Monthly Household Income	−0.0301*** (0.0105)
MWTP Altruism	£270	MWTP Altruism	£1140
No. Observations	7608	No. Observations	7608
R-Square	0.0804	R-Square	0.0190
Pre-treatment F-Statistic Test	2.017 [0.3648]	Pre-treatment F-Statistic Test	1.289 [0.5251]
Playing a Useful Role		Depression	
Transfer Made* Covid-19 Period	−0.0675** (0.0317)	Transfer Made* Covid-19 Period	−0.1439** (0.0630)
Logarithm of Monthly Household Income	−0.0691*** (0.0148)	Logarithm of Monthly Household Income	−0.1056*** (0.0201)
MWTP Altruism	£460	MWTP Altruism	£650
No. Observations	7608	No. Observations	7608
R-Square	0.0316	R-Square	0.0658
Pre-treatment F-Statistic Test	1.151 [0.5625]	Pre-treatment F-Statistic Test	0.3244 [0.8522]
Losing Confidence		Losing Confidence	
Transfer Made* Covid-19 Period	−0.0749** (0.0365)	Transfer Made* Covid-19 Period	−0.0977* (0.0582)
Logarithm of Monthly Household Income	−0.1131*** (0.0185)	Logarithm of Monthly Household Income	−0.1098*** (0.0186)
MWTP Altruism	£310	MWTP Altruism	£420
No. Observations	7608	No. Observations	7608
R-Square	0.0784	R-Square	0.0793
Pre-treatment F-Statistic Test	1.764 [0.4141]	Pre-treatment F-Statistic Test	0.3423 [0.8427]
Happiness		Believe Worthless	
Transfer Made* Covid-19 Period	−0.0383** (0.0181)	Transfer Made* Covid-19 Period	−0.0882* (0.0521)
Logarithm of Monthly Household Income	−0.0362*** (0.0131)	Logarithm of Monthly Household Income	−0.1195*** (0.0173)
MWTP Altruism	£500	MWTP Altruism	£350
No. Observations	7608	No. Observations	7608
R-Square	0.0235	R-Square	0.0791
Pre-treatment F-Statistic Test	1.769 [0.4129]	Pre-treatment F-Statistic Test	0.1008 [0.9508]
Treated (Transfers Made to Siblings) - White British		Treated (Transfers Made to Friends) - White British	
GHQ-12 Caseness		Losing Confidence	
Transfer Made* Covid-19 Period	−0.4237** (0.2099)	Transfer Made* Covid-19 Period	−0.0977* (0.0582)
Logarithm of Monthly Household Income	−0.4079*** (0.0761)	Logarithm of Monthly Household Income	−0.1098*** (0.0186)
MWTP Altruism	£490	MWTP Altruism	£420
No. Observations	7608	No. Observations	7608
R-Square	0.0810	R-Square	0.0793
Pre-treatment F-Statistic Test	0.0325 [0.9838]	Pre-treatment F-Statistic Test	2.314 [0.3145]
Treated (Transfers Made to Siblings) - White British		Treated (Transfers Made to Friends) - White British	
Playing a Useful Role		GHQ-12 Caseness	
Transfer Made* Covid-19 Period	−0.1191** (0.0571)	Transfer Made* Covid-19 Period	−0.0829** (0.0389)
Logarithm of Monthly Household Income	−0.0688*** (0.0142)	Logarithm of Monthly Household Income	−0.4058*** (0.0761)
MWTP Altruism	£820	MWTP Altruism	£90
No. Observations	7608	No. Observations	7608
R-Square	0.0319	R-Square	0.0806
Pre-treatment F-Statistic Test	1.252 [0.5348]	Pre-treatment F-Statistic Test	0.0664 [0.9673]
Constantly under strain		Ability to Face Problems	
Transfer Made* Covid-19 Period	−0.1463* (0.0817)	Transfer Made* Covid-19 Period	−0.1945* (0.1023)
Logarithm of Monthly Household Income	−0.0475** (0.0185)	Logarithm of Monthly Household Income	−0.0318*** (0.0104)
MWTP Altruism	£1400	MWTP Altruism	£1650
No. Observations	7608	No. Observations	7608
R-Square	0.0731	R-Square	0.0246
Pre-treatment F-Statistic Test	0.5028 [0.7774]	Pre-treatment F-Statistic Test	0.3806 [0.8267]
Problem Overcoming Difficulties		Believe Worthless	
Transfer Made* Covid-19 Period	−0.1290* (0.0779)	Transfer Made* Covid-19 Period	−0.1420** (0.0614)
Logarithm of Monthly Household Income	−0.0970*** (0.0184)	Logarithm of Monthly Household Income	−0.0458** (0.0185)
MWTP Altruism	£630	MWTP Altruism	£1470
No. Observations	7608	No. Observations	7608
R-Square	0.0550	R-Square	0.0735
Pre-treatment F-Statistic Test	0.0691 [0.9662]	Pre-treatment F-Statistic Test	2.537 [0.2812]
Depression		Happiness	
Transfer Made* Covid-19 Period	−0.1676** (0.0796)	Transfer Made* Covid-19 Period	−0.0386** (0.0182)
Logarithm of Monthly Household Income	−0.1085*** (0.0199)	Logarithm of Monthly Household Income	−0.0357*** (0.0137)
MWTP Altruism	£740	MWTP Altruism	£500
No. Observations	7608	No. Observations	7608
R-Square	0.0645	R-Square	0.0233
Pre-treatment F-Statistic Test	2.752 [0.2525]	Pre-treatment F-Statistic Test	0.1271 [0.9284]
Believe Worthless			
Transfer Made* Covid-19 Period	−0.1692** (0.0703)		
Logarithm of Monthly Household Income	−0.1223*** (0.0173)		
MWTP Altruism	£660		
No. Observations	7608		
R-Square	0.0783		
Pre-treatment F-Statistic Test	0.9564 [0.6199]		
Treated (Transfers Made to Parents-Grandparents)-Other White and White Mixed		Treated (Transfers Made to Siblings)- Other White and White Mixed	
GHQ-12 Caseness		GHQ-12 Caseness	
Transfer Made* Covid-19 Period	−1.8042** (0.7740)	Transfer Made* Covid-19 Period	−0.0720* (0.0359)
Logarithm of Monthly Household Income	−0.8035** (0.3550)	Logarithm of Monthly Household Income	−0.0301*** (0.0105)
MWTP Altruism	£1130	MWTP Altruism	£1200
No. Observations	716	No. Observations	716
R-Square	0.2067	R-Square	0.0190
Pre-treatment F-Statistic Test	2.403 [0.3008]	Pre-treatment F-Statistic Test	0.623 [0.7342]
Constantly Under Strain		Constantly Under Strain	
Transfer Made* Covid-19 Period	−0.5083** (0.2521)	Transfer Made* Covid-19 Period	−0.5227** (0.2481)
Logarithm of Monthly Household Income	−0.1366** (0.0670)	Logarithm of Monthly Household Income	−0.1443** (0.0678)
MWTP Altruism	£1800	MWTP Altruism	£1830
No. Observations	716	No. Observations	716
R-Square	0.2310	R-Square	0.2252
Pre-treatment F-Statistic Test	0.5027 [0.7274]	Pre-treatment F-Statistic Test	1.023 [0.5996]
Problem Overcoming Difficulties		Depression	
Transfer Made* Covid-19 Period	−0.3690* (0.2181)	Transfer Made* Covid-19 Period	−0.6679*** (0.2125)
Logarithm of Monthly Household Income	−0.1131*** (0.0185)	Logarithm of Monthly Household Income	−0.1935** (0.0863)
MWTP Altruism	£940	MWTP Altruism	£1750
No. Observations	716	No. Observations	716
R-Square	0.1832	R-Square	0.2281
Pre-treatment F-Statistic Test	1.132 [0.5678]	Pre-treatment F-Statistic Test	1.307 [0.5202]
Happiness		Believe Worthless	
Transfer Made* Covid-19 Period	−0.4349** (0.2053)	Transfer Made* Covid-19 Period	−0.5470** (0.2559)
Logarithm of Monthly Household Income	−0.1043** (0.0511)	Logarithm of Monthly Household Income	−0.1261** (0.0563)
MWTP Altruism	£1600	MWTP Altruism	£2190
No. Observations	716	No. Observations	716
R-Square	0.1788	R-Square	0.2104
Pre-treatment F-Statistic Test	0.0723 [0.9645]	Pre-treatment F-Statistic Test	1.0134 [0.6025]
Treated (Transfers Made to Friends)-Other White and White Mixed			
Ability to Face Problems		Believe Worthless	
Transfer Made* Covid-19 Period	−0.2674** (0.1256)	Transfer Made* Covid-19 Period	−0.4422** (0.2102)
Logarithm of Monthly Household Income	−0.1232*** (0.0379)	Logarithm of Monthly Household Income	−0.2063** (0.0804)
MWTP Altruism	£1050	MWTP Altruism	£1080
No. Observations	716	No. Observations	716
R-Square	0.2281	R-Square	0.2159
Pre-treatment F-Statistic Test	0.3487 [0.8402]	Pre-treatment F-Statistic Test	0.4127 [0.8136]
Treated (Transfers Made to Adult Children)-India, Pakistan, Bangladesh			
Enjoy day-to-day Activities		Believe Worthless	
Transfer Made* Covid-19 Period	−0.5195** (0.2449)	Transfer Made* Covid-19 Period	−0.4579* (0.2402)
Logarithm of Monthly Household Income	−0.1279** (0.1074)	Logarithm of Monthly Household Income	−0.2146** (0.0926)
MWTP Altruism	£2050	MWTP Altruism	£1090
No. Observations	1024	No. Observations	1024
R-Square	0.2023	R-Square	0.3830
Pre-treatment F-Statistic Test	1.354 [0.5082]	Pre-treatment F-Statistic Test	0.2511 [0.8821]
Treated (Transfers Made to Parents-Grandparents)- India, Pakistan, Bangladesh			
Losing Confidence		Happiness	
Transfer Made* Covid-19 Period	−0.6835** (0.3169)	Transfer Made* Covid-19 Period	−0.5274** (0.2161)
Logarithm of Monthly Household Income	−0.1970** (0.0885)	Logarithm of Monthly Household Income	−0.2160** (0.0924)
MWTP Altruism	£1770	MWTP Altruism	£1250
No. Observations	1024	No. Observations	1024
R-Square	0.3365	R-Square	0.2315
Pre-treatment F-Statistic Test	1.287 [0.5255]	Pre-treatment F-Statistic Test	0.4889 [0.7831]
Treated (Transfers Made to Siblings)- India, Pakistan, Bangladesh		Treated (Transfers Made to Friends)- India, Pakistan, Bangladesh	
Playing a Useful Role		Capable of Making Decisions	
Transfer Made* Covid-19 Period	−0.4494** (0.2206)	Transfer Made* Covid-19 Period	−0.3517** (0.1643)
Logarithm of Monthly Household Income	−0.1150** (0.0487)	Logarithm of Monthly Household Income	−0.1754** (0.0782)
MWTP Altruism	£2000	MWTP Altruism	£1370
No. Observations	1024	No. Observations	1024
R-Square	0.2655	R-Square	0.2447
Pre-treatment F-Statistic Test	1.2935 [0.5238]	Pre-treatment F-Statistic Test	0.3085 [0.8572]
Treated (Transfers Made to Adult Children)-Asians, Black Caribbean, Africans and Arabs			
Capable of Making Decisions		Enjoy day-to-day Activities	
Transfer Made* Covid-19 Period	−0.2920* (0.1529)	Transfer Made* Covid-19 Period	−0.3357** (0.1562)
Logarithm of Monthly Household Income	−0.1254** (0.0564)	Logarithm of Monthly Household Income	−0.1152** (0.0548)
MWTP Altruism	£1060	MWTP Altruism	£1350
No. Observations	924	No. Observations	924
R-Square	0.2181	R-Square	0.2015
Pre-treatment F-Statistic Test	0.5247 [0.7692]	Pre-treatment F-Statistic Test	1.175 [0.5558]
Treated (Transfers Made to Siblings)- Asians, Black Caribbean, Africans and Arabs			
Playing a Useful Role		Capable of Making Decisions	
Transfer Made* Covid-19 Period	−0.4501* (0.2503)	Transfer Made* Covid-19 Period	−0.2501** (0.1243)
Logarithm of Monthly Household Income	−0.1140** (0.0542)	Logarithm of Monthly Household Income	−0.1045** (0.0505)
MWTP Altruism	£1800	MWTP Altruism	£1100
No. Observations	924	No. Observations	924
R-Square	0.2171	R-Square	0.2119
Pre-treatment F-Statistic Test	0.9767 [0.6136]	Pre-treatment F-Statistic Test	0.4064 [0.8161]

Standard errors in the brackets and clustered at the individual level. P-values within the square brackets. ***, ** and * indicate significance at 1%, 5% and 10% level. Regressions are weighted by the sampling survey weight

Regarding the last two ethnic groups, which is those coming from an Indian, Pakistani and Bangladeshi background and those who are Asians, blacks from Caribbean, Africans and Arabs, we find that transfers and altruism impact a lower number of SWB measures. Regarding the first group and according to the *WTPA* values, respondents evaluate more their SWB from transfers made to family members than friends, as we found also in the case of the non-UK born whites and mixed whites. A similar concluding remark is derived from the analysis of the second group -Asians, black Caribbean, African and Arabs-, where the impact of altruism on SWB was found significant only in the transfers made to adult children and siblings.

## Conclusions

This study had two main objectives. The first aim was to investigate the impact of altruism on various SWB measures using a DiD framework and exploiting the Covid-19 lockdown measures in the UK. The second aim was to evaluate the willingness to pay for improvement in SWB due to altruistic behaviour, expressed by transfers made to adult children, parents-grandparents, siblings and friends. The findings suggest that there are no differences across gender and racial-ethnic groups based on the *WTPA* values, but there are differences in the SWB domains. In particular, we found that altruistic activities affect males’ capability of making decisions, and confidence, while for women we found that their happiness, depression and with daily activities are more important domains of their SWB. Similarly, regarding the analysis by racial and ethnic groups, we find similarities across all the groups, in terms of the influence of altruism on SWB measures, except for the group of Arabs, Africans, Asians and Black Caribbean, where altruism was found to affect only their capability of making decisions, playing a useful role and joy with daily activities.

Furthermore, apart from the SWB domains, we find also differences in the *WTPA* values across the groups of recipients. More specifically, men evaluate more their siblings and friends, while women are willing to pay more for altruistic acts and transfers made to parents-grandparents and adult children, to improve their SWB. Regarding the analysis on the ethnic and racial background, we found that white British evaluate more their SWB from transfers made to friends, compared to the remained ethnic groups that derive higher values from transfers made to family members.

Overall, as we have discussed earlier, previous studies found mixed results, as some support that women are more altruistic, including also recession periods, than men, while other studies found insignificant gender differences. Our findings suggest that there are no differences in the altruistic behaviour among gender and racial-ethnic groups, but there are differences in terms of the SWB domains and the willingness to support more a certain group of recipients.

However, the estimates and the argument about causal inference should be treated with caution. In particular, the Covid-19 pandemic may have increased income uncertainty for the giver or may have changed the living arrangements and generated large health and other adjustment costs that could potentially affect both subjective well-being and transfers.

## Data Availability

The data have been derived from the UK Data Service and are available for research purposes. This study uses data from the UK Household Longitudinal Study (UKHLS) in waves 7–9 and the UKHLS Covid-19 survey conducted in April 2020.
